# Determinants of Dropout From Correctional Offender Treatment

**DOI:** 10.3389/fpsyt.2019.00142

**Published:** 2019-03-22

**Authors:** Franziska Brunner, Insa Neumann, Dahlnym Yoon, Martin Rettenberger, Elisabeth Stück, Peer Briken

**Affiliations:** ^1^Institute for Sex Research and Forensic Psychiatry, University Medical Center Hamburg-Eppendorf, Hamburg, Germany; ^2^Institute of Psychology, FernUniversität in Hagen, Hagen, Germany; ^3^Centre for Criminology (Kriminologische Zentralstelle – KrimZ), Wiesbaden, Germany; ^4^Department of Psychology, Johannes Gutenberg-University (JGU), Mainz, Germany

**Keywords:** dropout, sexual offender, violent offender, correctional treatment, risk factor, protective factor, psychopathy

## Abstract

Research indicates that approximately one third of offenders admitted to social-therapeutic correctional facilities in Germany fail to complete treatment and that treatment dropout is linked to higher recidivism in both sexual and violent offenders. The purpose of this study was to examine determinants of treatment dropout in a social-therapeutic correctional facility in Germany. The sample consisted of 205 incarcerated adult male offenders (49.8% sexual, 38.1% non-sexual violent) admitted to correctional treatment. Completers and dropouts were compared on variables pertaining to demographics, offense type, substance abuse, psychopathy, risk, and protective factors. Univariate analyses showed that treatment dropouts demonstrated significantly higher scores on measures of risk and psychopathy and lower scores on protective factors. Logistic regression analyses identified unemployment, non-sexual violent index offense, higher risk scores (HCR-20), and Facet 1 (interpersonal deficits) of the Psychopathy Checklist-Revised (PCL-R) as significant predictors of treatment dropout. Surprisingly, substance abuse disorder was a negative predictor of dropout. With the exception of substance abuse, the results support the notion that treatment dropouts represent a group of high-risk offenders with particular treatment needs. Practical implications and suggestions for further research are discussed.

In Germany, legislation regulates that social therapy represents the primary form of correctional treatment in prisons for sexual offenders whose sentencing is for more than two years (German Federal Penal Execution Law §9). Additionally, non-sexual offenders can apply for social therapy. Previous research indicates that approximately one third of offenders admitted to social-therapeutic correctional facilities in Germany fail to complete treatment (1). Comparable results were noted in an international meta-analysis by Olver et al. (2), who reported an overall attrition rate of 27.6% in sexual offender programs (*k* = 34, *n* = 12,878) and 26.9% in non-sexual violent offender programs (*k* = 9, *n* = 1,238). These numbers for several reasons raise concerns. First, those who do not complete treatment are unlikely to derive its benefits, posing a potential risk to public safety. In fact, research has shown that treatment dropout is linked to higher recidivism risk in both sexual and violent offenders (2–4). In a systematic review, McMurran and Theodosi (4) presented evidence that program dropout might even increase the risk of reoffending compared to receiving no treatment at all. Second, research has repeatedly shown that especially high-risk high-need offenders [in terms of the risk, need, responsivity model by Bonta and Andrews (5)] are those who are less likely to complete treatment, calling for effective interventions to retain these individuals in treatment (2, 6). Third, treatment dropout has negative economic implications if resources are misallocated to participants who are unlikely to gain from the program and waitlisted offenders remain untreated (7). Fourth, dropout poses a problem for evaluation studies assessing the effectiveness of treatment programs. Excluding treatment dropouts from these studies, as it is common practice, can lead to potential overestimations of treatment effects, demonstrating the need for more elaborate research designs to permit more accurate evaluation of program effects including dropouts (8–10). Given its possible detrimental effects, research is required to identify factors associated with treatment dropout and to develop measures as well as therapeutic techniques to promote treatment completion.

Research identifying predictors associated with treatment dropout has yielded inconsistent results. In part, the outcomes are so divergent that Larochelle et al. (11) concluded that “it is difficult to draw unequivocal conclusions about the variables related to the phenomenon (p. 554)”. Nevertheless, meta-analytic reviews identified numerous variables associated with treatment dropout across different treatment programs: With regard to demographic variables, higher rates of treatment dropout were associated with single marital status, lower educational attainment, higher unemployment rates, lower income, younger age, and ethnic minority status (2, 12). Moreover, dropouts tend to have higher rates of prior offenses and incarcerations and shorter sentence lengths; prior violent offenses were more strongly related to treatment attrition than prior non-violent offenses when compared across different treatment programs (2, 12). Static risk assessment instruments, which base their prediction predominantly on offense-related variables, predicted treatment dropout across different treatment programs, particularly in sexual offenders (2, 6, 13). Evidence on clinical variables suggests that substance abuse is linked to treatment dropout. However, results differ with regard to offender groups. Whereas a significant association between treatment dropout and substance abuse was found for domestic violence programs (2, 12, 14), no relationship was reported for sexual offender treatment (2). Moreover, higher rates of psychopathy have repeatedly been linked to higher treatment dropout rates, and in turn to higher rates of violent recidivism (15–17).

Whereas there is a strong evidence base linking risk factors to treatment dropout and recidivism risk, protective factors, which could retain individuals in treatment, have received less scholarly attention. A review in the field of domestic violence treatment suggested that programs designed to enhance motivation for changes and to address individual needs, such as personality traits, could decrease treatment attrition (18). A small body of empirical research has additionally shown that absent substance abuse, employment, and intimate relationship were positively related to treatment completion [i.e., (19)]. Recent studies have found that a decrease in dynamic risk and an increase in protective factors during treatment predicted reductions in recidivism (20, 21). Therefore, evaluating both risk and protective factors in the course of treatment could enhance treatment completion and outcomes (22, 23).

## Social-Therapeutic Treatment in Germany

Admission to social-therapeutic correctional facilities is regulated by Art. 9 of the German Federal Penal Execution Code (StVollzG; Strafvollzugsgesetz). For sexual offenders who serve a minimum 2-year prison sentence, admission to a social-therapeutic correctional facility is mandatory. Sexual offenders shall only be transferred back to a general correctional facility if the purpose of the treatment cannot be achieved for reasons inherent in the person of the prisoner. Non-sexual offenders may apply for admission to the social-therapeutic correctional facility on their own initiative. Their admission requires the approval of the management. According to the federal law, admission should be granted if the institution's special therapeutic means and social assistance are appropriate for their resocialization. The therapeutic concept of the social-therapeutic institution of the federal state Hamburg suggests that, in addition to the need for treatment, responsivity factors (such as sufficient German language skills or introspection capability) and the motivation of the offender are decisive for the selection of the non-sexual offenders. In practice, however, there may be deviations due to the occupancy situation in Hamburg prisons and non-sexual offenders may be admitted who do not fully meet these criteria. Social-therapeutic treatment has no fixed length. Legislation allows a transfer to general prison if an offender is unlikely to generate treatment gains. Social therapy is characterized by a progressive transfer of responsibility to the client and the promotion of social learning within the community. Integrative social therapy follows three core principles (24): (1) consideration and inclusion of the offenders' living environment within and outside the social-therapeutic correctional facility until release; (2) development of opportunities and relationships within the social-therapeutic correctional facility in terms of a therapeutic community; (3) modification and integration of approaches based in psychotherapy, pedagogy, and occupational therapy. Within the program, participants receive the opportunity to take part in a variety of offers, such as vocational training, education, work opportunities, or individual and group psychotherapy.

Nationwide, social-therapeutic correctional facilities display heterogeneity regarding the kinds of interventions they offer (25). Besides psychodynamic-oriented milieu therapy, the social-therapeutic correctional facility in the present study offered both offense-specific group therapy, such as the Sex Offender Treatment Program [SOTP; (26–28)], strength-based approaches for sexual offender rehabilitation (29), and general and offense-unspecific group treatments, covering topics such as substance abuse and addiction. Additionally, participants can receive individual therapy sessions, special interventions, and support for release planning.

## Study Aim

The purpose of the present study was twofold. First, it sought to identify relevant variables related to treatment dropout among offenders in a social-therapeutic correctional facility in Germany. The variables under study pertained to demographic and offense characteristics, recidivism risk, psychopathy, and protective factors. Based on the findings reviewed above, it was expected that treatment dropouts would more likely be single (never married), less educated (no secondary school diploma), unemployed, non-German, younger, and non-sexual violent offenders. They would suffer from substance abuse, demonstrate higher levels of risk and psychopathy, and score lower on protective factors than those who completed treatment. Second, the study explored whether any of the empirical-driven variables were predictive of treatment dropout. The rationale behind this was the creation of a model allowing the identification of offenders at increased risk of dropping out of the specialized treatment for sexual and violent offenders in a social-therapeutic correctional facility.

## Materials and Methods

### Procedure

This study was part of a large research project “Evaluation of the Social-Therapeutic Correctional Facility Hamburg” (30), which was authorized and funded by the Ministry of Justice of the Free and Hanseatic City of Hamburg. The ongoing research project is being conducted by the Institute for Sex Research and Forensic Psychiatry at the University Medical Center Hamburg-Eppendorf (UKE) since 2010. The study was approved by the ethical committee of the Hamburg chamber of psychotherapists. Participants were informed about the purpose of the research project and gave their written informed consent in accordance with the Declaration of Helsinki. The aim was a complete survey of all offenders (only men) serving sentences at the social-therapeutic correctional facility of the Hamburg correctional services (SothA-HH). In the survey period from 2010 to 2018, all new entrants were informed about the study. Thirty-eight announced inmates (18.5%) refused to participate.

Data of the present study was based on pretreatment ratings and collected on site within the first weeks after the participants' admission to the SothA-HH. All data were derived from case file information (e.g., criminal record, court files, or psychological reports) and from semi-structured interviews, which lasted approximately 2 hours per participant. Information about treatment dropout was provided by the SothA-HH administration. All data were collected by trained psychologists.

### Participants

The participants were *N* = 205 male offenders serving sentences at SothA-HH between the years 2010 and 2018. Social-therapeutic correctional treatment had been indicated and had started for all included participants. *Completion* was defined as either (a) having participated in treatment programs of the social-therapeutic correctional facility for at least three years or (b) having been released or (c) having been regularly transferred to another facility. Participants who did not complete social-therapeutic treatment and were consequently transferred back to general prison were classified as *dropouts*. Dropout status was determined irrespective of whether the dropout was initiated by the offender himself or SothA-HH staff. Offender type was determined based on the index offenses the participants were currently detained for. The “other” group refers to offenders who committed neither sexual nor non-sexual violent offenses but other crimes (e.g., fraud or theft). A more in-depth analysis of the criminological and risk assessment characteristics of the sample can be found in Brunner et al. (30).

### Measures

#### Demographic and Offense Variables

Demographic variables in the study were marital status (ever married vs. never married), education (secondary school diploma vs. no secondary school diploma), employment prior to incarceration (employed or student/trainee vs. neither employed nor student/trainee), nationality (German vs. non-German), and age at time of the data collection. Offense type (sexual vs. non-sexual violent vs. other) was defined based on the index offense participants were currently detained for. Substance abuse (yes vs. no) was defined as lifetime mental and behavioral disorder due to psychoactive substance use [ICD-10 criteria for harmful use or dependency syndrome; (31)]. Index offense sentence length (months) was ascertained by court files. In case of accompanying sentences, only the index offense sentence was taken into account, unless a court has formed an overall penalty for several single convictions. Lifetime sentences were counted as 300 months. Preventive detentions were not considered in this variable.

#### Psychopathy Checklist-Revised (PCL-R)

The PCL-R (32, 33) is a 20-item measure of psychopathic personality traits. The instrument was designed with two interrelated factors, which are further divided into two facets each. The facets subsumed under Factor 1 describe interpersonal (Facet 1) and affective deficits (Facet 2). Factor 2 pertains to chronic antisocial behavior, and its facets are impulsive lifestyle (Facet 3) and antisocial behavior (Facet 4). Each variable is scored on a 3-point scale (0–2) with total scores ranging from 0 to 40. Based on Hare (33), scores on the PCL-R can be categorized into three levels, with values between 0 and 16 indicating a low score, values between 17 and 24 indicating a medium score, and values above 24 indicating a high score on the construct. The reliability, concurrent and predictive validity of the PCL-R have been supported by a substantial body of literature (33–37). In case of omitted items, prorated scores were used.

#### Historical Clinical Risk Management-20 (HCR-20)

The HCR-20 [(38); German version: (39)] is a widely used structured professional judgment (SPJ) instrument for the assessment of risk for violent (including sexual violent) behavior. The tool comprises 20 static and dynamic variables, divided into ten historical (e.g., previous violence, young age at first violent incident, employment problems), five clinical (e.g., lack of insight, negative attitudes, impulsivity), and five risk management factors (e.g., lack of personal support, non-compliance with remediation attempts, stress). Each item is scored on a 3-point scale (0–2), and the rater assigns a structured final risk judgment (low, medium, or high). The instrument has demonstrated good concurrent validity (40) and moderate to strong predictive accuracy (41, 42) in offender populations. In the present study, all offender groups (including sexual offenders) were assessed with the HCR-20 second version.

#### Structured Assessment of PROtective Factors for Violence Risk (SAPROF)

The SAPROF [(43); German version: (44)] is an SPJ instrument assessing protective factors reducing violent risk. It is used in combination with SPJ risk assessment instruments, such as the HCR-20. The checklist contains 17 protective factors, with two static and 15 dynamic variables. Factors are organized into *internal factors, motivational factors*, and *external factors*. Internal factors refer to personal characteristics with protective benefits (e.g., intelligence, empathy, self-control), motivational factors assess an individual's motivation to become a positive member of society (e.g., work, motivation for treatment, attitude towards authority), and external items comprise social, judicial and therapeutic control factors (e.g., social network, intimate relationships, external control). All items are rated on a 3-point scale (0–2) and a protection and an integrated risk level (low, medium, or high) is assigned, taking the combined judgment of the SAPROF and HCR-20 into account. In a sample of forensic psychiatric patients, the instrument demonstrated good inter-rater reliability and good predictive validity for non-recidivism of (sexual) violence in forensic psychiatric patients (45, 46). The German version of the SAPROF has shown small to moderate predictive accuracy regarding sexual offenders recidivism in the correctional system (47).

### Statistical Analyses

Univariate analyses were applied to identify relevant differences between dropouts and completers. More specifically, χ^2^-tests were used for categorical variables and one-way analyses of variance (ANOVA) for continuous variables, the effect sizes were calculated by Cramer's *V* and η^2^. A Bonferroni-Holm correction was applied over all univariate tests in order to reduce the risk of alpha-error cumulation. All variables that were identified as relevant predictors for treatment attrition in previous studies and had been possible to assess in the present research project were entered into the logistic regression model with treatment completion status as the binary outcome variable. According to this empirical-driven procedure, the following variables were entered into the model: offender type, marital status, education, unemployment, nationality, substance abuse, age at admission, the HCR-20 sum score, all four facets of the PCL-R, and the SAPROF sum score. HCR-20 and SAPROF subscales were not entered separately to circumvent power loss due to a large model size. In a second data-driven approach, the model with the best fit was identified via stepwise backward elimination per likelihood-ratio-test (48). Data analyses were performed using IBM SPSS Statistics 23 software.

## Results

### Sample Characteristics

**Figure 1:**
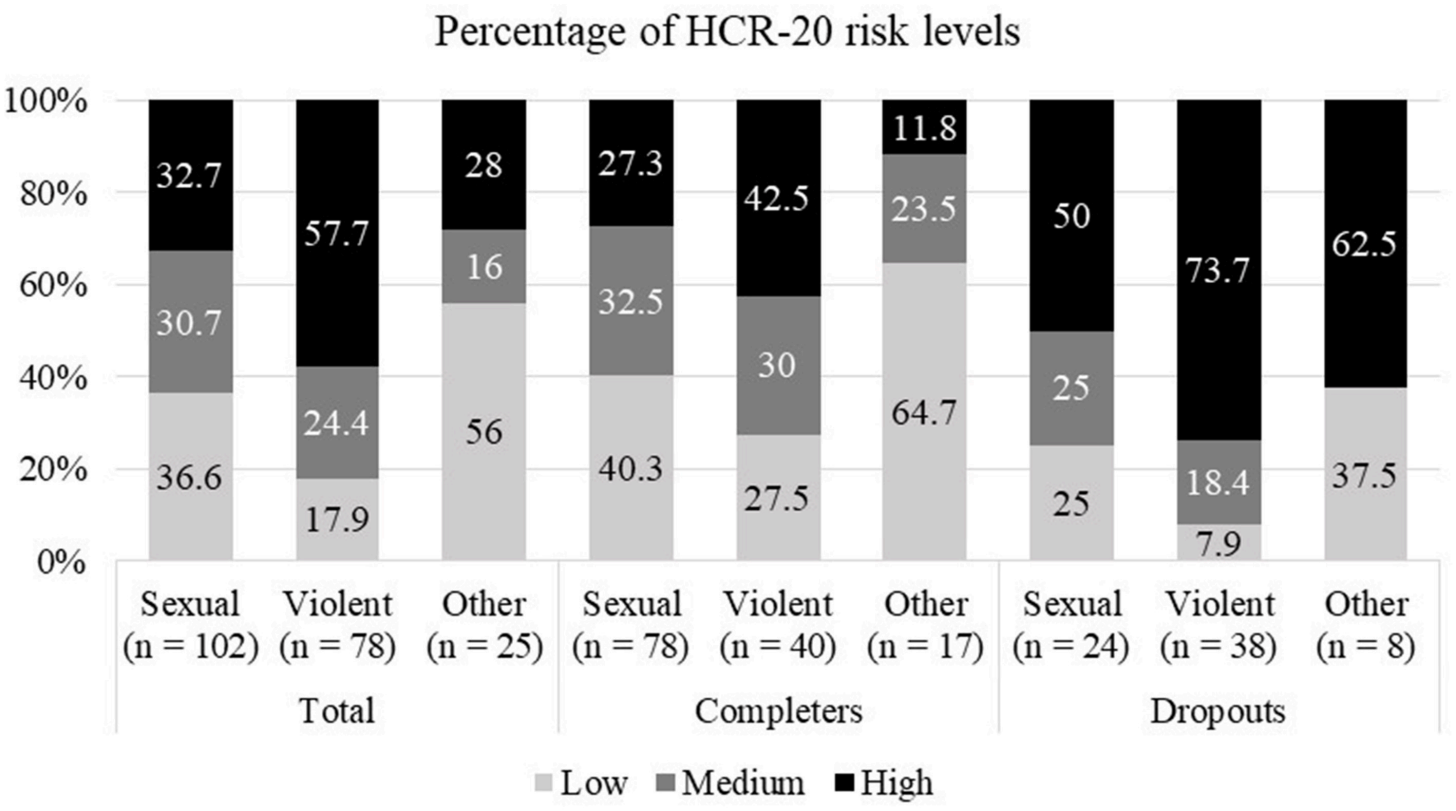
Relative distribution of HCR-20 risk levels for offender group and dropout status.

The sample comprised *N* = 205 male inmates. Since all sexual offenders were transferred to the SothA-HH with a prison sentence of over 2 years, this group accounts for the largest offender group with 49.8%. Non-sexual violent offenders are represented in the sample by 38.0% and others by 12.2%. Overall, 70 participants (34.1%) were classified as dropouts and 135 (65.9%) as completers. Figure 1 shows how the risk of renewed violent crimes differs between offender groups. Among sex offenders, the lowest risk category accounts for the largest share at 36.6%, while 57.7% of violent offenders fall into the highest risk category. Table 1 shows an overview of the sample's demographic and criminological characteristics. Educational attainment ranged from no general education at all (27.3%; *n* = 56), secondary education (70.7%; *n* = 145) to tertiary education (2.0%; *n* = 4).

**Table 1:** Demographic and criminological characteristics.

**Variable**	***n***	**%**	***M ± SD***	**Range**
Offender type	205	100.0		
Sexual	102	49.8		
Violent	78	38.0		
Other	25	12.2		
Never married	116	56.6		
No education	56	27.3		
Unemployment	98	47.8		
Non-German	87	42.4		
Substance abuse	115	56.1		
Age at admission (years)	205		36.60 ± 11.52	21–67
Age at first conviction (years)	205		24.26 ± 11.54	13–65
Age at first incarceration (years)	200		28.85 ± 12.02	14–67
Number of prior convictions	205		6.65 ± 6.60	0–26
Index offense sentence length (months)^a^	205		57.41 ± 47.88	7–300

*The variables “never married” and “age at first incarceration” contained n = 1 and n = 5 cases of missing data, respectively*.

a *Life sentences were counted as 300 months*.

### Offender Type and Demographics

Table 2 shows frequencies of completion and dropout for offender type and demographics. A 2 × 3 χ^2^-test indicated that the relationship between completion status and offender group was significant. The majority of dropouts (54.3%; *n* = 38) was incarcerated for a non-sexual violent index offense. In contrast, within the completer group (*n* = 135), non-sexual violent index offenses made up only 29.6% (*n* = 40) of the index offenses. Completers were predominantly incarcerated for sexual offenses (57.8%; *n* = 78), whereas this offense type accounted for about a third of offenses among dropouts (34.3%; *n* = 24). Frequencies of other offenses were roughly similar in both groups. Further, univariate analyses yielded significant group differences for unemployment at the time of incarceration.

**Table 2:** Comparisons of completers (*n* = 135) and dropouts (*n* = 70) regarding offender type and various demographics.

	**Completers**		**Dropouts**					
**Variable**	***n***	**% or *M ± SD***	***n***	**% or *M ± SD***	**χ^2^ or F**	***df***	***P***	***V* or η^**2**^**
**OFFENDER TYPE**								
Sexual	78	57.8	24	34.3	12.53		**0.002**	0.25
Violent	40	29.6	38	54.3		2		
Other	17	12.6	8	11.4				
**DEMOGRAPHICS**								
Never married	76	56.7	40	57.1	<0.01	1	1.00	<0.01
No education	29	21.5	27	38.6	6.78	1	0.013	0.18
Unemployed	53	39.3	45	64.3	11.57	1	**0.001**	0.24
Non-German	56	41.5	31	44.3	0.15	1	0.766	0.03
Substance abuse	70	51.9	45	64.3	2.89	1	0.103	0.12
Age	135	37.85 ± 12.30	70	34.17 ± 9.46	4.80	1.203	0.030	0.02

*Bold values indicate significance after Bonferroni-Holm correction. The variable “never married” contained n = 1 case of missing data*.

### Risk Factors

In Tables 3, 4, the results of statistical analyses of risk (HCR-20), psychopathy (PCL-R) and protective factors (SAPROF) are presented. Dropouts scored significantly higher on the HCR-20 and its respective subscales compared to treatment completers (Table 3). This finding is also confirmed by the χ^2^-Test indicating a significant association between completion status and HCR-20 risk levels (Table 4); the dropout group consisted of a relatively higher proportion of high-risk offenders. Conversely, low and medium risk levels were reported for the majority of completers (70.2%; *n* = 94), but only for a minority of dropouts (35.7%; *n* = 25).

**Table 3:** Comparisons of completers (*n* = 134) and dropouts (*n* = 70) regarding risk and protective factors.

	**Completers**	**Dropouts**				
**Variable**	***M ± SD***	***M ± SD***	***F***	***df***	***p***	**η^**2**^**
**RISK FACTORS**						
HCR-20	17.07 ± 6.41	22.87 ± 6.50	37.33	1.202	**<0.001**	0.16
Historical	8.78 ± 4.03	12.17 ± 3.88	33.31	1.202	**<0.001**	0.14
Clinical	3.25 ± 1.79	4.67 ± 2.01	26.41	1.202	**<0.001**	0.12
Risk	5.03 ± 1.66	6.03 ± 1.76	15.92	1.202	**<0.001**	0.07
PCL-R	14.20 ± 6.52	20.86 ± 7.18	44.84	1.202	**<0.001**	0.18
Facet 1: interpersonal deficits	1.97 ± 1.85	3.06 ± 2.35	13.20	1.202	**<0.001**	0.06
Facet 2: affective deficits	3.49 ± 1.81	4.39 ± 2.02	10.41	1.202	**0.001**	0.05
Facet 3: impulsive lifestyle	4.12 ± 2.35	6.11 ± 2.29	33.67	1.202	**<0.001**	0.14
Facet 4: antisocial behavior	3.42 ± 2.84	5.79 ± 3.26	28.86	1.202	**<0.001**	0.13
**PROTECTIVE FACTORS**						
SAPROF	15.40 ± 3.51	13.93 ± 3.66	7.79	1.202	0.006	0.04
Internal	4.43 ± 1.34	3.57 ± 1.54	17.14	1.202	**<0.001**	0.08
Motivational	5.01 ± 2.07	4.46 ± 1.83	3.51	1.202	0.063	0.02
External	5.96 ± 1.18	5.90 ± 1.12	0.11	1.202	0.746	0.00

*Bold values indicate significance after Bonferroni-Holm correction. Each of the variables contained n = 1 case of missing data, but PCL-R Facet 4 contained n = 2 cases of missing data. HCR-20, Historical Clinical Risk Assessment-20; PCL-R, Psychopathy Checklist-Revised; SAPROF, Structured Assessment of PROtective Factors*.

**Table 4:** Pearson χ2-test of completion status by risk and protection levels.

	**Completers**		**Dropouts**		**Total**						
**Measure**	***n***	**%**	***n***	**%**	***n***	**%**	***n***	**χ^2^**	***df***	***p***	***V***
**HCR-20 (*****n*** **= 204)**											
Low	53	39.6	12	17.1	65	31.9	204	22.84		**<0.001**	0.34
Medium	41	30.6	13	18.6	54	26.5			2		
High	40	29.9	45	64.3	85	41.7					
**PCL-R (*****n*** **= 204)**											
Low	85	63.4	21	30.0	106	52.0	204	34.23		**<0.001**	0.41
Medium	42	31.3	26	37.1	68	33.3			2		
High	7	5.2	23	32.9	30	14.7					
**SAPROF (*****n*** **= 203)**											
Low	49	36.6	40	58.0	89	43.8	203	9.43		0.009	0.22
Medium	62	46.3	24	34.8	86	42.4			2		
High	23	17.2	5	7.2	28	13.8					

*Bold values indicate significance after Bonferroni-Holm correction. The HCR-10 risk and PCL-R risk judgment contained n = 1 case of missing data each. The SAPROF protection judgment contained n = 2 cases of missing data. HCR-20 = Historical Clinical Risk Assessment-20; PCL-R, Psychopathy Checklist-Revised; SAPROF, Structured Assessment of PROtective Factors*.

As indicated by Table 3, completers and dropouts differed significantly on psychopathy, with dropouts scoring significantly higher compared to completers. Analyses on facet level confirmed this finding for PCL-R Factors 1 and 2; dropouts scored significantly higher than completers on PCL-R Facets 1 and 2 as well as on PCL-R Facets 3 and 4. A χ^2^-test indicated significant disproportional frequencies between psychopathy level and completion status. Dropouts tended to have increased PCL-R scores, with approximately one third of this group reaching the cut-off of 25. In comparison, only 5.2% (*n* = 7) of completers scored high on psychopathy. Nearly one third of dropouts (30.0%; *n* = 21) received a low PCL-R score. In contrast, approximately twice the proportion of completers (63.4%; *n* = 85) scored low on the degree of psychopathic personality traits.

### Protective Factors

As reported in Table 3, completers and dropouts differed significantly on the internal SAPROF subscale, with dropouts scoring significantly lower compared to completers. The χ^2^-test (Table 4) indicated no significant relationship between completion status and level of protection after Bonferroni-Holm correction (*p*_adj_ = 0.006). Overall, the majority of offenders (86.2%; *n* = 175) received low or medium protection levels. Most dropouts (58.0%; *n* = 40) scored low on protection, whereas among the completers, the medium protection levels accounted for the largest share with 46.3% (*n* = 62). In addition, the data showed that approximately twice the proportion of completers received high protection ratings, compared to dropouts.

### Logistic Regression Analyses

As described above, previously reported predictors of treatment attrition were entered into a logistic regression with treatment completion status as the binary outcome variable. Table 5 shows that this first model significantly predicted treatment completion and based on Nagelkerke's *R*^2^, explained 36% of the pseudo-variation. Compared to the constant alone, the overall model improved the prediction of completion status by 12.4% (from 65.3 to 77.7%). Analysis of the individual contributions of the predictors showed violent offender type, substance abuse, and PCL-R Facet 1 emerged as significant predictors of treatment dropout, when all other predictors were held constant.

**Table 5:** Logistic regression analysis predicting treatment dropout–first model (*N* = 202).

			**95% CI**	
**Measure**	***OR***	***p***	***LL***	***UL***
Offender type: sexual		0.051		
Offender type: violent	2.52	**0.024**	1.13	5.61
Offender type: other	0.95	0.932	0.30	3.01
Never married	0.60	0.250	0.25	1.44
No education	1.15	0.734	0.52	2.53
Unemployment	2.05	0.058	0.98	4.31
Non-German	1.76	0.135	0.84	3.70
Substance abuse	0.30	**0.012**	0.12	0.77
Age	0.99	0.535	0.95	1.03
HCR-20 sum	1.10	0.102	0.98	1.23
PCL-R Facet 1: interpersonal deficits	1.27	**0.016**	1.05	1.53
PCL-R Facet 2: affective deficits	0.97	0.760	0.77	1.21
PCL-R Facet 3: impulsive lifestyle	1.20	0.145	0.94	1.54
PCL-R Facet 4: antisocial behavior	1.05	0.553	0.89	1.25
SAPROF sum	1.02	0.720	0.90	1.16
Constant	0.02	**0.037**		

*Nagelkerke R^2^ = 0.36 (n = 3 cases were reported missing and excluded from analysis). Bold values indicate significance at p ≤ 0.05. OR, Odds Ratio; CI, confidence interval; LL, lower limit; UL, upper limit; HCR-20, Historical Clinical Risk Assessment-20; PCL-R, Psychopathy Checklist-Revised; SAPROF, Structured Assessment of PROtective Factors*.

**Figure 2:**
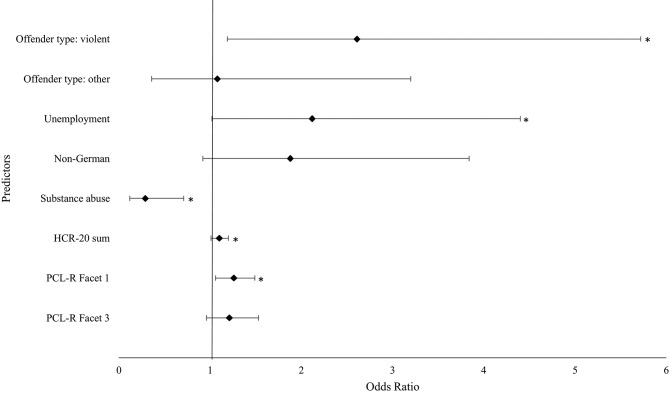
Predictors for the treatment dropout of the model with the best fit. *indicates significance at *p* ≤ 0.05. CI, confidence interval; HCR-20, Historical Clinical Risk Assessment-20; PCL-R, Psychopathy Checklist-Revised.

In order to identify the model with the best fit, a stepwise backward elimination per likelihood-ratio-test was conducted (see Table 6, Figure 2). Overall, the new model correctly classified 74.8% of the cases and explained 35% of pseudo-variation (Nagelkerke's *R*^2^). Interpretation on the variable level showed that violent offender type, unemployment, substance abuse, HCR-20 sum score, and PCL-R Facet 1 significantly predicted treatment dropout.

**Table 6:** Logistic regression predicting treatment dropout–model with best fit after stepwise backward elimination per likelihood-ratio-test (*N* = 202).

			**95% CI**	
**Measure**	***OR***	***p***	***LL***	***UL***
Offender type: sexual		**0.044**		
Offender type: violent	2.61	**0.017**	1.19	5.72
Offender type: other	1.08	0.893	0.36	3.20
Unemployment	2.12	**0.044**	1.02	4.40
Non-German	1.88	0.083	0.92	3.84
Substance abuse	0.29	**0.008**	0.12	0.72
HCR-20 sum	1.10	**0.023**	1.01	1.20
PCL-R Facet 1: interpersonal deficits	1.26	**0.008**	1.06	1.49
PCL-R Facet 3: impulsive lifestyle	1.21	0.102	0.96	1.53
Constant	0.01	**0.000**		

*Nagelkerke R2 = 0.35 (n = 3 cases were reported missing and excluded from analysis). Bold values indicate significance at p ≤ 0.05. OR, Odds Ratio; CI, confidence interval; LL, lower limit; UL, upper limit; HCR-20, Historical Clinical Risk Assessment-20; PCL-R, Psychopathy Checklist-Revised; SAPROF, Structured Assessment of PROtective Factors*.

## Discussion

The current study examined determinants of treatment dropout in a male offender sample undergoing treatment in a social-therapeutic correctional facility in Germany. First, dropouts and completers were compared on several demographic, criminogenic risk and protective variables. Second, empirical-driven predictor variables were entered into two logistic regression models predicting treatment dropout. Several findings emerged from the analyses.

### Finding 1: Admission of Medium to High-Risk Offenders for Social-Therapy but High Dropout Rate of High-Risk/High-Need Offenders

Risk estimates based on the HCR-20 scores indicated that medium to high-risk offenders serving sentences for sexual and non-sexual violent crimes are the typical clientele of social-therapeutic treatment. Especially among non-sexual violent offenders the proportion of high-risk offenders seemed particularly high compared to the sexual offender group. Having in mind that only non-sexual offenders may be selected before admission to the social-therapeutic facility, the overrepresentation of high-risk (non-sexual) violent offenders may indicate that social-therapeutic resources are indeed allocated to those who need them most [according to the RNR-model by Bonta and Andrews (5)]. However, analyses revealed that dropouts from social-therapeutic treatment demonstrated significantly higher levels on both recidivism risk and psychopathy measures. Therefore, a relatively high number of those offenders with high risk and high need for treatment could not be kept in therapy. These findings are in line with previous research demonstrating that non-completers are high-risk and high-need individuals and that psychopaths were proportionally overrepresented in groups of treatment or program dropouts (15, 17).

### Finding 2: Univariate Analyses Yielded Unemployment, Risk Factors and Internal Protective Factors as Significantly Different Between Completers and Dropouts

Except for unemployment, the univariate analyses yielded no significant differences between dropouts and completers on demographic variables and substance abuse. Recent studies have indeed found significant relationships between these variables and treatment dropout [i.e., (2)]. However, the lack of concordance with earlier research is not surprising, as an absence of consistent findings seems eminent to the field of attrition research (11) and may be explicable by differences in risk levels, types of treatment programs, populations under study, or ways in which dropout was operationalized. Motivational and external protective factors as assessed by the SAPROF did not significantly differentiate between the two groups. In line with the prediction, increased dropout rates were found among those who were unemployed, incarcerated for violent offenses, and scored high on risk (HCR-20), and psychopathy (all four facets of the PCL-R). These findings corroborate previous research showing that unemployment, violent offenses, risk and psychopathy were consistently associated with dropout (2).

Moreover, those with higher protection scores on the SAPROF internal subscale exhibited lower dropout rates. The higher manifestation of internal resources such as self-control, coping skills, intelligence, or empathy in the completer group might indicate that these factors are important prerequisites for treatment adherence. For example, research indicates that internal attributes like intelligence and self-control positively influence psychosocial adjustment and are able to prevent antisocial behavior (49, 50). While protective factors are still understudied, currently published research suggests risk-reducing effects on recidivism (23, 47) and that improvements in the domain of protection may also translate into reductions in treatment dropout rates (20).

### Finding 3: PCL-R Facet 1, Violent Index Offense, Unemployment, Substance Abuse, and HCR-20 Sum Score Are Predictors for Dropout

The model with the best fit after stepwise backward elimination per likelihood-ratio-test indicated five variable as significant predictors of treatment dropout: violent index offense, unemployment, substance abuse, HCR-20 sum score, and PCL-R Facet 1 (interpersonal deficits). Surprisingly, substance abuse was inversely related to treatment dropout. Each predictor will be discussed in more detail below.

#### PCL-R Facet 1

Offenders with high psychopathic traits are particularly challenging to treat because they represent an offender group that responds poorly to treatment, displays low motivation and disruptive behaviors, and has usually high treatment dropout rates (15, 16, 51, 52). Their treatment requires special attention as some programs might even hinder a positive therapy outcome [e.g., (53, 54)]. In the present study, PCL-R Facet 1 (interpersonal deficits) emerged as a significant predictor of treatment dropout. This finding suggests that the interpersonal problems (e.g., pathological lying, manipulative behavior, and a grandiose sense of self-worth) presented in persons with high psychopathic traits may be responsible for treatment dropout. Multiple reasons may be discussed. These offenders may have more problems to establish meaningful relationships compared to offenders with low or medium scores. In a study by Olver and Wong (16) higher scores on the PCL-R Facet 2 (affective deficits) significantly predicted treatment dropout in a sample of sexual offenders. The authors argued that affective deficits may impede the formation of strong therapeutic bonds. Arguably, this can be posited for both interpersonal problems *and* affective deficits. Inmates with interpersonal deficits subsumed under PCL-R Facet 1 are exhausting and unpleasant in contact and can deteriorate the atmosphere of the facility. These interpersonal deficits may thus be harmful to the establishment of a strong therapeutic alliance as they undermine mutual trust. The relationship between patient and therapist is known to be an important factor to achieve positive treatment outcomes (55). In a sample of sexual offenders, DeSorcy et al. (56) showed that lower ratings of working alliance were related to higher rates of treatment dropout, whereas some studies did not confirm this relationship (57). Further research is needed to investigate therapeutic alliance in psychopaths, since the relationship between psychopathy and dropout can be moderated by treatment alliance.

Another reason for the elevated dropout rates among offenders with higher psychopathic traits may be explained by higher rates of behavioral problems. In a sample of 44 high-risk offenders admitted to a forensic psychiatric hospital, PCL-R Facet 1 and 2 significantly predicted interpersonal physical aggression (58). The findings suggest that scoring high on PCL-R Factor 1 increases the likelihood to engage in violent behavior. This in turn may translate into increased back-transfer to general prison if the institution worries that an offender poses a danger to fellow inmates.

O'Brien and Daffern (52) investigated the role of psychopathy in treatment dropout in an Australian violent offender sample. The authors found that psychopathy moderated the level of treatment participation and violent reoffending: offenders with high psychopathy scores, who engaged with treatment or completed it, had similar violent recidivism rates compared to those offenders with low psychopathy scores. In contrast, those who scored high on the construct but engaged poorly in treatment or did not complete it demonstrated higher rates of violent recidivism. The abovementioned findings have important implications, as appropriate interventions and successfully retaining psychopathic offenders in treatment appeared to be related to therapeutic improvement and reduced risk of sexually and violently reoffending (17). Findings by Olver et al. (59) further indicate that positive therapeutic change is negatively related to PCL-R Factor 1 supporting a growing body of literature that suggests psychopathy may be treatable after all (60) and that Factor 1-related risk factors provide good treatment targets to reduce dropout.

#### Violent Offense

Being incarcerated for a non-sexual violent index offense significantly predicted treatment dropout. This finding was in line with previous research showing that prior violent offenses were related to increased treatment dropout and recidivism across treatment programs (2). Unlike sexual offenders, violent offenders are not automatically admitted to social therapy but must undergo an application process—although deviations due to the occupancy situation in Hamburg prisons are possible. It is likely that, among the violent offender applicants, the SothA-HH purposefully selected those with the highest risk status. A rationale behind the selection of high-risk offenders may be that the latter group has the highest need for treatment [cf. RNR-model; (5)]. The results showed that it remained difficult to retain non-sexual violent offenders in treatment, emphasizing the need for future research to study responsivity issues as avenues for interventions (5) to mitigate the risk for treatment dropout. These may include ways of motivational interviewing, low-threshold group interventions for preparation of specific therapy or very individualized forms of single therapy if there are sufficient resources. Due to the steadily increasing proportion of non-sexual violent offenders in the last few years (25), research about new developments and improvements of treatment programs as well as techniques particularly devised for non-sexual violent offenders is warranted.

#### Unemployment

Previous studies showed that employment instability/unemployment was predictive of both treatment dropout and recidivism (12, 61, 62). Whereas unemployment per se is unlikely to *cause* treatment attrition, it may be part of a larger pattern of lifestyle instability and antisocial behavior, as also evidenced by group differences on Facets 3 and 4 of the PCL-R. It is plausible that those individuals unable to keep a job will probably show more interpersonal problems as well as a less stable therapeutic commitment as both make similar demands on the individual such as regular attendance, responsibility, the acceptance of rules and authority, and display of pro-social behavior. Thus, an individual who previously quit or lost his jobs frequently due to impulsive, irresponsible, rule-violating, or aggressive behavior may display similar behavior in a therapeutic context, which is likely to result in the premature termination of treatment. Based on these considerations, a specific targeting of criminogenic needs such as self-control, anger issues, or lack of perseverance may provide positive improvements for both employability and treatment outcomes. Moreover, treatment approaches based on the Good Lives Model (GLM) would focus on employment and education issues, in order to equip individuals with the capabilities to achieve outcomes which were considered as desired and beneficial by the majority of the society (63). Tentative findings by Ullrich and Coid (23) as well as Yoon et al. (47) suggest that under certain circumstances, employment could act as a protective factor reducing the risk of reoffending.

#### Substance Abuse

Substance abuse emerged as a significant predictor of treatment dropout, but, paradoxically, was inversely related to the criterion variable: offenders who had a diagnosis of substance abuse were *less* likely to drop out of social-therapeutic treatment. This is remarkable considering that substance abuse is a risk factor and has previously repeatedly been linked to treatment dropout in violent offenders (2, 12, 14). In fact, substance abuse are especially difficult to treat and dropout rates from treatment programs for substance abuse are oftentimes higher than 50% (64). In drug abuse treatment programs, dropout is actually considered a risk factor, as it increases the likelihood of a relapse (65). Similar to the present results, the meta-analysis of Olver et al. (2) found a small negative correlation between substance use problems and sex offender treatment dropout (*r*_*w*_ = −0.04), albeit this trend was not significant. Additionally, a study with 126 incarcerated sexual offenders also found that treatment completers were more likely to suffer from substance use disorder (66). The divergence in findings between sexual and violent offenders suggests that the relationship between dropout and substance abuse may be modulated by offender group. At present, we can only speculate why substance abuse is inversely related to treatment dropout. The finding may be explained by an increased allocation of resources to offenders with substance abuse. Being known as high-risk and difficult-to-treat individuals, offenders with substance abuse issues may have received additional treatment offers and were treated with particular attention to their needs. For example, the inmates of SothA-HH have access to an additional treatment for offenders with substance abuse. Future research is needed to investigate the role of substance abuse in predicting treatment dropout.

#### HCR-20 Sum Score

Every predictor for dropout already discussed is a component of the HCR-20: Therefore, an index as well as prior violent offense, psychopathic traits, substance abuse, and employment instability may contribute to a high risk indicated by HCR-20 sum score. Additionally, high HCR-20 sum scores can indicate clinical risk factors such as lack of insight, antisocial, and hostile attitudes or impulsivity, but also risk factors such as noncompliance and an antisocial environment that make a future without renewed violent delinquency unlikely. At the same time, all these factors probably contribute in part to making it more difficult to cooperate with and adapt to a social-therapeutic correctional facility.

### Limitations

Several limitations should be noted and addressed by future research. First, data on the reasons for dropout could not be obtained. This could threaten the validity of the results, if participants who exit the SothA-HH due to a systemic factor, such as administrative transfer, were accidentally categorized as dropouts. It could render interpretation of the results difficult, as dropout due to administrative reasons cannot be explained in terms of offender characteristics or behavior but rather external circumstances beyond the offender's control. Despite this being theoretically problematic, exits due to systemic factors happen only rarely in practice and their number in the present sample should be negligible. Future research would benefit from more detailed information on dropout reasons as they could provide a better understanding of the nature of treatment attrition and its relationship to the independent variables under investigation. Second, the generalizability of the findings is limited to the present population. Although, the participation rate is with 81.5% satisfactory (especially for a prisoners' sample), we cannot exclude self-selection bias resulting from refusers. Moreover, cross-validation with a different sample is advised when assessing the model's performance in practice. This is of particular importance, as social-therapeutic treatment is distinct to the German penal system, posing a threat to external validity if transferring results to international contexts. Finally, the current study could not investigate if dropout from a social-therapeutic facility did in fact translate into the assumed higher recidivism rates. Future research should test this hypothesis to reach a better understanding of the relationships between diverse risk and protective factors, dropout, and recidivism risk.

## Conclusion

Despite some limitations, the present study provides important insights into the relationship between numerous variables and treatment dropout. The results support the notion that dropouts represent a high-risk and high-need offender group with pronounced risk and psychopathy scores, violent offense histories, and higher unemployment rates. Violent index offense, unemployment at the time of incarceration, HCR-20 sum score, PCL-R Facet 1, and, surprisingly, absence of substance abuse disorder were identified as significant predictors of treatment dropout, raising important considerations for treatment practice. Further research is necessary to determine how these variables contribute to treatment dropout, and to examine which variables exert a possibly confounding influence on the relationship between unemployment and treatment dropout. Even though findings regarding the relationship between dropout and protective factors remain inconclusive, further research should investigate if reductions in treatment dropout may be achieved if programs were adapted to address strengths as well as deficits.

## Author Contributions

FB, IN, and PB designed the study. FB, DY, and MR collected the data. FB, IN, and ES analyzed and interpreted the data. IN wrote the initial draft of the manuscript in constant consultation with FB. FB, IN, ES, and PB had full access to all the data in the study and take responsibility for the integrity of the data and the accuracy of data analysis. All authors have contributed to, read, and approved the final version of the manuscript.

### Conflict of Interest Statement

The authors declare that the research was conducted in the absence of any commercial or financial relationships that could be construed as a potential conflict of interest. The reviewer KD declared a past collaboration with one of the authors DY to the handling editor.

## References

[1] Spöhr M Sozialtherapie von Sexualstraftätern im Justizvollzug: Praxis und Evaluation [Social Therapy of Sexual Offenders in Law Enforcement: Practice and Evaluation]. Mönchengladbach: Forum Verlag (2009).

[2] Olver ME Stockdale KC Wormith JS. A meta-analysis of predictors of offender treatment attrition and its relationship to recidivism. J Consult Clin Psychol. (2011) 79:6. 10.1037/a0022200 21261430

[3] Hanson RK Bussiere MT. Predicting relapse: a meta-analysis of sexual offender recidivism studies. J Consult Clin Psychol. (1998) 66:348–62. 10.1037/0022-006X.66.2.348 9583338

[4] McMurran M Theodosi E Is treatment non-completion associated with increased reconviction over no treatment? Psychol Crime Law. (2007) 13:333–43. 10.1080/10683160601060374

[5] Bonta J Andrews DA The Psychology of Criminal Conduct. New York, NY: Routledge (2016).

[6] Wormith JS Olver ME Offender treatment attrition and its relationship with risk, responsivity, and recidivism. Crim Justice Behav. (2002) 29:447–71. 10.1177/0093854802029004006

[7] Polaschek DLL Treatment non-completion in high-risk violent offenders: looking beyond criminal risk and criminogenic needs. Psychol Crime Law. (2010) 16:525–40. 10.1080/10683160902971048

[8] Endres J Breuer MM Stemmler M „Intention to treat“ oder „treatment as received“ – Umgang mit Abbrechern in der Forschung zur Straftäterbehandlung [Intention-to-treat and treatment-as-received: dealing with dropouts in research on offender treatment]. Forensisc Psychiatr Psychol Kriminol. (2016) 10:45–55. 10.1007/s11757-015-0348-x

[9] Hatcher RM McGuire J Bilby CAL Palmer EJ Hollin CR. Methodological considerations in the evaluation of offender interventions: the problem of attrition. Int J Offender Ther Comp Criminol. (2011) 56:447–64. 10.1177/0306624X11403271 21518700

[10] Rice ME Harris GT. The size and sign of treatment effects in sex offender therapy. Ann NY Acad Sci. (2003) 989:428–40. 10.1111/j.1749-6632.2003.tb07323.x 12839916

[11] Larochelle S Diguer L Laverdière O Greenman PS. Predictors of psychological treatment noncompletion among sexual offenders. Clin Psychol Rev. (2011) 31:554–62. 10.1016/j.cpr.2010.12.004 21239099

[12] Jewell LM Wormith JS Variables associated with attrition from domestic violence treatment programs targeting male batterers: a meta-analysis. Crim Justice Behav. (2010) 37:1086–113. 10.1177/0093854810376815

[13] Nunes KL Cortoni F Dropout from sex-offender treatment and dimensions of risk of sexual recidivism. Crim Justice Behav. (2008) 35:24–33. 10.1177/0093854807309037

[14] Daly JE Pelowski S. Predictors of dropout among men who batter: a review of studies with implications for research and practice. Violence Vict. (2000) 15:137–60. 10.1891/0886-6708.15.2.137 11108498

[15] Ogloff JRP Wong SCP Greenwood A Treating criminal psychopaths in a therapeutic community program. Behav Sci Law. (1990) 8:181–90. 10.1002/bsl.2370080210

[16] Olver ME Wong SCP Predictors of sex offender treatment dropout: psychopathy, sex offender risk, and responsivity implications. Psychol Crime Law. (2011) 17:457–71. 10.1080/10683160903318876

[17] Olver ME Wong SCP. Therapeutic responses of psychopathic sexual offenders: treatment attrition, therapeutic change, and long-term recidivism. J Consult Clin Psychol. (2009) 77:328–36. 10.1037/a0015001 19309191

[18] Sartin RM Hansen DJ Huss MT Domestic violence treatment response and recidivism: a review and implications for the study of family violence. Aggress Violent Behav. (2006) 11:425–40. 10.1016/j.avb.2005.12.002

[19] Gover AR Jennings WG Davis C Tomsich EA Tewksbury R Factors related to the completion of domestic violence offender treatment: the colorado experience. Vict Offender. (2011) 6:137–56. 10.1080/15564886.2011.557323

[20] de Vries Robbe M de Vogel V Douglas KS Nijman HL. Changes in dynamic risk and protective factors for violence during inpatient forensic psychiatric treatment: predicting reductions in postdischarge community recidivism. Law Hum Behav. (2015) 39:53–61. 10.1037/lhb0000089 24933171

[21] Tozdan S Briken P Yoon D von Franqué F Risiko- und Schutzfaktoren bei sexualdelinquent gewordenen Menschen: Vorhersage erneuter Straftaten und Veränderungen im Behandlungsverlauf [Risk and protective factors among sexual offenders: relapse prediction and changes during treatment]. Psychiatr Prax. (2016) 43:154–9. 10.1055/s-0034-1387404 25526503

[22] de Ruiter C Nicholls TL Protective factors in forensic mental health: a new frontier. Int J Forensic Ment Health. (2011) 10:160–70. 10.1080/14999013.2011.600602

[23] Ullrich S Coid J. Protective factors for violence among released prisoners: effects over time and interactions with static risk. J Consult Clin Psychol. (2011) 79:381. 10.1037/a0023613 21500887

[24] Arbeitskreis Sozialtherapeutische Anstalten im Justizvollzug e V Sozialtherapeutische Anstalten und Abteilungen im Justizvollzug. Mindestanforderungen an Organisation und Ausstattung sowie Indikation zur Verlegung – Revidierte Empfehlungen (Stand 2016). (Stand 2016). Forum Strafvollzug Strafvollzug Straffälligenhilfe. (2016) 65:37–40.

[25] Etzler S Sozialtherapie im Strafvollzug 2017: Ergebnisübersicht zur Stichtagserhebung zum 31.03.2017 (BM-Online, Bd. 12). Wiesbaden: Kriminologische Zentralstelle (2017). Available online at: https://www.krimz.de/fileadmin/dateiablage/E-Publikationen/BM-Online/bm-online12.pdf

[26] Friendship C Mann RE Beech AR. Evaluation of a national prison-based treatment program for sexual offenders in England and Wales. J Interpers Violence. (2003) 18:744–59. 10.1177/0886260503253236 14675507

[27] Grubin D Thornton D A national program for the assessment and treatment of sex offenders in the English prison system. Crim Justice Behav. (1994) 21:55–71. 10.1177/0093854894021001005

[28] Mann RE Thornton D The evolution of a multisite sexual offender treatment program. In: Marshall WL Fernandez YM Hudson SM Ward T, editors. Sourcebook of Treatment Programs for Sexual Offenders. Boston, MA: Springer (1998). p. 47–57.

[29] Marshall WL Marshall LE Serran GA O'Brien MD Rehabilitating Sexual Offenders: A Strength-Based Approach. Washington, DC: American Psychological Association (2011).

[30] Brunner F Yoon D Rettenberger M Briken P Kriminologische und kriminalprognostische Merkmale der Insassen der Sozialtherapeutischen Anstalt Hamburg [Criminological and risk assessment characteristics of inmates in the social-therapeutic institution of the Hamburg correctional services]. Recht Psychiatr. (2016) 34:221–7.

[31] World Health Organization [WHO] The ICD-10 Classification of Mental and Behavioural Disorders: Clinical Descriptions and Diagnostic Guidelines, Vol. 1 Geneva: World Health Organization (1992).

[32] Hare RD Manual for the Hare Psychopathy Checklist-Revised. Toronto: Multi-Health Systems (1991).

[33] Hare RD The Psychopathy Checklist - Revised. Toronto: Multi-Health Systems (2003).

[34] Dahle KP. Strengths and limitations of actuarial prediction of criminal reoffence in a German prison sample: a comparative study of LSI-R, HCR-20 and PCL-R. Int J Law Psychiatry. (2006) 29:431–42. 10.1016/j.ijlp.2006.03.001 16780950

[35] Eher R Rettenberger M Hirtenlehner H Schilling F Dimensionale Struktur und prognostische Relevanz der PCL-R in einer Population österreichischer Sexualstraftäter [Factorial structure and predictive validity of the PCL-R in an Austrian sexual offender population]. Monatsschr Kriminol. (2012) 95:235–51.

[36] Hare RD Clark D Grann M Thornton D. Psychopathy and the predictive validity of the PCL-R: an international perspective. Behav Sci Law. (2000) 18:623–45. 10.1002/1099-0798(200010)18:5<623::AID-BSL409>3.0.CO;2-W 11113965

[37] Stadtland C Hollweg M Kleindienst N Dietl J Reich U Nedopil N Rückfallprognosen bei Sexualstraftätern – Vergleich der prädiktiven Validität von Prognoseinstrumenten [Prediction of recidivism in sexual offenders – comparison of the predictive validity of prognosis instruments]. Nervenarzt. (2006) 77:587–95. 10.1007/s00115-005-1945-2 15965760

[38] Webster CD Douglas KS Eaves D Hart SD HCR-20: Assessing the Risk for Violence (version 2). Vancouver: Mental Health, Law, and Policy Institute, Simon Fraser University (1997).

[39] Müller-Isberner R Jöckel D Gonzalez Cabeza S Die Vorhersage von Gewalttaten mit dem HCR-20 [The Prediction of Violent Offenses with the HCR-20]. Haina: Institut für Forensische Psychiatrie Haina (1998).

[40] Douglas KS Webster CD The HCR-20 violence risk assessment scheme: concurrent validity in a sample of incarcerated offenders. Crim Justice Behav. (1999) 26:3–19. 10.1177/0093854899026001001

[41] Douglas KS Yeomans M Boer DP Comparative validity analysis of multiple measures of violence risk in a sample of criminal offenders. Crim Justice Behav. (2005) 32:479–510. 10.1177/0093854805278411

[42] Yang M Wong SCP Coid J. The efficacy of violence prediction: a meta-analytic comparison of nine risk assessment tools. Psychol Bull. (2010) 136:740–67. 10.1037/a0020473 20804235

[43] de Vogel V de Ruiter C Bouman Y de Vries Robbé M SAPROF. Guidelines for the Assessment of Protective Factors for Violence Risk. English version. Utrecht: Forum Educatief (2009).

[44] Spehr A Briken P SAPROF. Leitlinien für die Erfassung von Protektiven Faktoren bei einem Risiko für Gewalttätiges Verhalten [SAPROF. Guidelines for the Measurement of Protective Factors in Case of Risk for Violent Behavior]. Utrecht: Forum Educatief (2010).

[45] de Vries Robbé M de Vogel V de Spa E Protective factors for violence risk in forensic psychiatric patients: a retrospective validation study of the SAPROF. Int J Forensic Ment Health. (2011) 10:178–86. 10.1080/14999013.2011.600232

[46] de Vries Robbé M de Vogel V Koster K Bogaerts S. Assessing protective factors for sexually violent offending with the SAPROF. Sex Abuse. (2015) 27:51–70. 10.1177/1079063214550168 25210106

[47] Yoon D Turner D Klein V Rettenberger M Eher R Briken P. Factors predicting desistance from reoffending: a validation study of the SAPROF in sexual offenders. Int J Offender Ther Comp Criminol. (2018) 62:697–716. 10.1177/0306624X16664379 27531703

[48] Vittinghoff E McCulloch CE. Relaxing the rule of ten events per variable in logistic and Cox regression. Am J Epidemiol. (2007) 165:710–8. 10.1093/aje/kwk052 17182981

[49] Kandel E Mednick SA Kirkegaard-Sorensen L Hutchings B Knop J Rosenberg R. IQ as a protective factor for subjects at high risk for antisocial behavior. J Consult Clin Psychol. (1988) 56:224–6. 10.1037/0022-006X.56.2.224 3372829

[50] Tangney JP Baumeister RF Boone AL. High self-control predicts good adjustment, less pathology, better grades, and interpersonal success. J Pers. (2004) 72:271–324. 10.1111/j.0022-3506.2004.00263.x 15016066

[51] Hobson J Shine J Roberts R How do psychopaths behave in a prison therapeutic community? Psychol Crime Law. (2000) 6:139–54. 10.1080/10683160008410838

[52] O'Brien K Daffern M The impact of pre-treatment responsivity and treatment participation on violent recidivism in a violent offender sample. Psychol Crime Law. (2016) 22:777–97. 10.1080/1068316X.2016.1181177

[53] Swogger MT Conner KR Caine ED Trabold N Parkhurst MN Prothero LM. A test of core psychopathic traits as a moderator of the efficacy of a brief motivational intervention for substance-using offenders. J Consult Clin Psychol. (2016) 84:248. 10.1037/ccp0000065 26727409 PMC4760863

[54] Wong SCP Hare RD Guidelines for a Psychopathy Treatment Program. Toronto: Multi-Health Systems (2005).

[55] Martin DJ Garske JP Davis MK. Relation of the therapeutic alliance with outcome and other variables: a meta-analytic review. J Consult Clin Psychol. (2000) 68:438–50. 10.1037/0022-006X.68.3.438 10883561

[56] DeSorcy DR Olver ME Wormith JS. Working alliance and its relationship with treatment outcome in a sample of aboriginal and non-aboriginal sexual offenders. Sex Abuse. (2014) 28:291–313. 10.1177/1079063214556360 25381308

[57] Walton A Jeglic EL Blasko BL. The role of psychopathic traits in the development of the therapeutic alliance among sexual offenders. Sex Abuse. (2016) 30:211–29. 10.1177/1079063216637859 27000265

[58] Langton CM Hogue TE Daffern M Mannion A Howells K. Personality traits as predictors of inpatient aggression in a high-security forensic psychiatric setting: prospective evaluation of the PCL-R and IPDE dimension ratings. Int J Offender Ther Comp Criminol. (2011) 55:392–415. 10.1177/0306624X10370828 20463208

[59] Olver ME Lewis K Wong SCP. Risk reduction treatment of high-risk psychopathic offenders: the relationship of psychopathy and treatment change to violent recidivism. Pers Disord Theory Res Treat. (2013) 4:160. 10.1037/a0029769 23046041

[60] Salekin RT Psychopathy and therapeutic pessimism: clinical lore or clinical reality? Clin Psychol Rev. (2002) 22:79–112. 10.1016/S0272-7358(01)00083-6 11793579

[61] Gendreau P Goggin C Gray G Case Needs Review: Employment Domain (Research Report No. R-90). Ottawa, ON: Correctional Service of Canada (2000).

[62] Hanson RK Morton-Bourgon KE. The characteristics of persistent sexual offenders: a meta-analysis of recidivism studies. J Consult Clin Psychol. (2005) 73:1154–63. 10.1037/0022-006X.73.6.1154 16392988

[63] Ward T Mann RE Gannon TA The good lives model of offender rehabilitation: clinical implications. Aggress Violent Behav. (2007) 12:87–107. 10.1016/j.avb.2006.03.004

[64] Brorson HH Ajo Arnevik E Rand-Hendriksen K Duckert F. Drop-out from addiction treatment: a systematic review of risk factors. Clin Psychol Rev. (2013) 33:1010–24. 10.1016/j.cpr.2013.07.007 24029221

[65] Hser Y-I Evans E Huang D Anglin DM. Relationship between drug treatment services, retention, and outcomes. Psychiatr Serv. (2004) 55:767–74. 10.1176/appi.ps.55.7.767 15232015

[66] Moore DL Bergman BA Knox PL Predictors of sex offender treatment completion. J Child Sex Abuse. (1999) 7:73–88. 10.1300/J070v07n03_05

